# Assessment of Quality of Life Among the Geriatric Population in an Urban Slum Settlement of Bhubaneswar, Odisha

**DOI:** 10.7759/cureus.43664

**Published:** 2023-08-17

**Authors:** Avinandan Sarkar, Ipsa Mohapatra, Rabindranath Rout

**Affiliations:** 1 Community Medicine, Kalinga Institute of Medical Sciences, Kalinga Institute of Industrial Technology (KIIT) University, Bhubaneswar, IND

**Keywords:** aging, who-bref, urban slum, quality of life, elderly

## Abstract

Background: Multidimensional components of quality of life such as well-being, satisfaction, and lifestyle together ensure an improvement in longer life expectancy. Therefore, it is important to emphasize the assessment of the quality of life (QoL) of the elderly population and its association with various background characteristics.

Methodology: A community-based cross-sectional study was carried out from April to September 2017 among 270 consenting elderly people residing in urban slums under the field practice area of a medical college, using a well-structured, globally validated World Health Organization Quality of Life Brief Version (WHOQOL-BREF) tool. The scores were computed for the different domains of WHO QoL, and the mean score of these domains was compared among the different socio-economic and demographic factors.

Results: The majority (67.41%) of the sampled population belonged to the 60- to 69-year-old age group; females (63.70%) outnumbered males. The total QoL score was 47.52 ± 15.06 showing that the average population had a fair QoL. The environmental domain had a poor QoL (43.15) score, and the rest of the domains showed a fair QoL. Higher age groups, persons other than self-employed, and the upper class were seen to have significantly lower QoL scores in all four domains. Females had a lower QoL score in the physical as well as social domains as compared to males.

Conclusion: Aging is associated with a decrease in social and economic participation. In areas of compromised living conditions, like slums, the environmental domain adds to the decrease in quality along with the other domains. Ageing, employment status, socio-economic class, and the female gender had significantly lower QoL scores in all four domains.

## Introduction

Health and quality of life (QoL) are interlinked. As life expectancy continues to rise, one of the greatest challenges for public health is improving QoL in the later years of life. Quality of life is a holistic approach that not only emphasizes an individual's physical, psychological, and spiritual functioning but also their connections with the environment and opportunities for maintaining and enhancing skills. The WHO has defined QoL as "an individual's perception of life in the context of culture and value system in which he or she lives and in relation to his or her goals, expectations, standards and concerns" [1].

The epidemiological transition of diseases with an increase in the burden of chronic morbidity conditions driven by population aging affects the QoL of the elderly population. As there are a multiplicity of morbidities and functional disabilities in old age, geriatric care places a considerable burden on the health system and family members. One of the major determinants of the QoL of elderly subjects is their functional status and the individual's ability to live independently, relate to his/her environment, or perform normal daily activities for basic needs and carry out normal functions to maintain health and wellbeing [2].

The QoL can be influenced by the social environment and living conditions. Rapid urbanization and societal modernization have brought in their wake a breakdown in family values and the framework of family support, economic insecurity, social isolation, and elderly abuse, leading to a host of psychological illnesses [3].The world is experiencing a change in the age structure, with an increase in life expectancy and decreasing levels of fertility, thereby leading to a rise in the number of older people. In 2020, the number of people aged more than 65 years was 727 million globally, and this is expected to reach more than 1.5 billion over the next three decades by 2050 [4].

Urban slums being areas of compromised living conditions with poor, unhygienic environments, the QoL of the elderly is expected to not be up to adequate standards. The current study was an attempt to assess QoL and its associated factors in different domains among the geriatric population in an urban slum settlement in Bhubaneswar, Odisha, India.

## Materials and methods

A cross-sectional study was done among the elderly population (≥ 60 years old) residing in the urban slums of Bhubaneswar, Odisha, India, from April to September 2017. The slums taken up for the study were in the field practice area of the Urban Health and Training Centre (UHTC), under the Department of Community Medicine, Kalinga Institute of Medical Sciences. The field practice area comprises five slums, covering a total population of about 12,500 with 3120 households.

All individuals aged ≥ 60 years of age who gave informed written consent were included. Participants who were unable to provide information were excluded. Persons who were deaf/mute/blind, those with diagnosed psychiatric illness (schizophrenia, mental retardation) or neurological disorders (Parkinsonism, severe head injury, or brain neoplasm), and those who were ill (any acute episode of illness) at the time of the study were also excluded, as it would not be possible to obtain reliable information from them.

The sample size was calculated by applying the sample-size formula: n = z2 pq/ d2 [5]. Where n = the sample size to be estimated; z = the standard normal deviate set as 1.96; p = the estimated proportion of problem (assuming the health morbidity in elderly persons aged 60 and above as 20% [6]); q = 1-p and d = the precision (0.05). Taking an additional 10% for non-response, the optimum sample size was taken as 270 for the study. A systematic random sampling method was applied to identify the households with eligible subjects, and further simple random sampling was used to identify one eligible subject from each household with more than one eligible subject. The study was approved by the Institutional Ethics Committee of the Institute (approval no. KIIT/KIMS/IEC/ 113/2015).

The study tool was a researcher-made schedule with three sections: socio-demographic (age, gender, educational status, occupation, religion, type of family, total number of members in the family), economic characteristics (socio-economic class based on the updated B J Prasad socioeconomic classification, 2014) [7], and WHOQOL-BREF (English version) questionnaire [1,8-10]. According to QoL scores, a total score of 110-89 is considered excellent, 8867 is good, 66-45 is fair, and 44-22 is poor [6]. The study tool was translated into Odia, the local language, by one expert and translated back into English by another expert to check the consistency of the original meaning of the WHOQOL-BREF questionnaire items. Furthermore, a meaningful modification in the content was done in consensus with three experts. The scale was again pilot tested (among 30 study participants) in a slum different from the study area, and the necessary changes were incorporated.

Data collection

Data was collected by the principal investigator and team (the authors of the article). All data were collected, coded, and tabulated using Microsoft Excel (Microsoft Corp., Redmond, WA, USA), and the analysis was carried out by the standard statistical software Stata 15.1 (StataCorp LLC, College Station, Texas, USA). Scores were computed for the different domains of WHO QoL, and the mean score of these domains was compared among the different socio-economic and demographic factors. Categorical variables were presented by frequency and percentage, and continuous variables, including all scores, were presented as mean ± standard deviation (SD). The mean score between the two groups was compared using an independent t-test, and it was compared between more than two groups by using one-way analysis of variance. The comparison between the two categorical variables was done using the chi-square test. A p-value of less than 0.05 was considered statistically significant.

## Results

A total of 270 elderly people, aged 60 years and older, were included and assessed for their QoL and its associated risk factors. Table 1 shows a summary of the socio-demographic and economic characteristics of the study subjects. Among the total subjects, 67.4% belonged to the age-group of 60 to 69 years, 36.3% were males, and 78.9% were Hindus.

**Table 1:** Socio-demographic and economic characteristics of the study subjects (n=270) *None belonged to other religions such as Christianity, Sikhism, etc. ^$^According to the BG Prasad 2014 socio-economic status scale SC: Scheduled caste, ST: Scheduled tribe

Characteristics	Classification	Frequency (percentage)
Age group (in years)	60-69	182 (67.41)
	70-79	67 (24.81)
	≥80	21 (7.78)
Sex	Male	98 (36.30)
	Female	172 (63.70)
Religion*	Hindu	213 (78.89)
	Muslim	57 (21.11)
Caste	SC/ST	29 (10.74)
	Other backward classes	65 (24.07)
	General	176 (65.19)
Marital status	Married	181 (67.04)
	Single	35 (12.96)
	Widow//widower/separated	54 (20.00)
Type of Family	Joint	54 (20.00)
	Nuclear	216 (80.00)
Educational status	Illiterate	48 (17.78)
	Primary school	200 (74.07)
	High school and above	22 (8.15)
Socio-economic status scale^$^	Upper class	86 (31.85)
	Upper middle class	9 (3.33)
	Middle class	9 (3.33)
	Lower middle class	65 (24.08)
	Lower class	101 (37.41)

According to the QoL scores, a total score of 110-89 is considered excellent, 88-67 is good, 66-45 is fair, and 44-22 is poor. In the present study, the total QoL score was 47.52 ± 15.06, showing that the average population had a fair QoL (Table 2). Specific to the environmental domain, the mean score of 43.15 is suggestive of poor QoL, and the rest of the domains showed a fair QoL.

**Table 2:** Summary of QoL assessment for different domains among the study subjects QoL: Quality of life

Domain	QoL score (Mean±SD)
Physica	52.74 ±13.36
Psychological	48.16 ±13.74
Social	46.03 ±19.5
Environmental	43.15 ±13.63
Total	47.52 ±15.06

Table 3 shows the association of various socio-demographic and economic characteristics with specific domains of QoL. Higher age groups, persons other than self-employed, and the upper class were seen to have significantly lower QoL scores in all four domains. Females had a lower QoL score in the physical as well as social domains as compared to males. The Hindu religion was shown to have a lower score in social and environmental domains as compared to Muslims. Persons who were either single or widow/widower/separated were seen to have lower QoL scores in the physical, social, and environmental domains. Socio-economic status was also significantly associated with all domains of QoL.

**Table 3:** Association of various socio-demographic and economic characteristics with different domains of QoL * signifies a statistically significant p-value QoL: Quality of life, SC: Scheduled caste, ST: Scheduled tribe, OBC: Other backward class

Characteristics	Physical	Psychological	Social	Environmental
Sex				
Male	54.62 ±13.75	48.55 ±14.3	48.18 ±19.4	43.36 ±14.09
Female	50.9 ±12.62	47.9 ±13.34	44.09 ±19.44	43.14 ±13.41
p-value	0.005^*^	0.35	0.04^*^	0.44
Age Group				
60 – 69	54.14 ±12.95	49.59 ±12.7	48.13 ±19.34	44.56 ±12.68
70 - 79	49.37 ±13.6	45.4 ±14.79	39.35 ±19.68	39.811 ±14.8
≥80	45.3 ±14.2	37.25 ±18.4	42.66 ±16.5	35.41 ±17.88
p value	0.001^*^	0.0003^*^	0.007^*^	0.002^*^
Religion				
Hindu	53.26 ±13.5	48.53 ±13.84	45.09 ±19.78	42.72 ±13.86
Muslim	52.95 ±12.2	49.5 ±13.86	54.35 ±21.70	48.65 ±13.77
p-value	0.40	0.38	0.03^*^	0.03^*^
Caste				
SC/ST	53.53 ±14.42	45.96 ±15.8	53.16 ±18.18	43.07 ±13.05
OBC	49.48 ±12.56	45.71 ±11.84	43.89 ±18.24	41.16 ±12.2
General	53.66 ±13.35	49.3 ±13.5	45.93 ±20.8	43.47 ±14.2
p-value	0.08	0.09	0.12	0.40
Marital status				
Married	54.38 ±13.3	48.77 ±13.36	50.2 ±17.2	44.86 ±13.34
Single	49.33 ±15.18	42.66 ±11.41	20.25 ±20.78	25 ±11.29
Widow/Widower/Separated	50.04 ±12.9	47.20 ±14.49	37.16 ±21.7	40.58 ±13.39
p-value	0.01^*^	0.38	0.0001^*^	0.0001^*^
Type of Family				
Joint	54.17 ±13.91	49.34 ±14.71	48.93 ±17.97	44.17 ±13.78
Nuclear	51.88 ±12.65	47.53 ±12.22	44.27 ±20.58	42.77 ±13.01
p-value	0.05	0.11	0.02^*^	0.17
Educational status				
Illiterate	51.59 ±11.63	48.42 ±13.11	46.09 ±16.67	43.05 ±12.60
Primary School	52.40 ±13.56	47.98 ±13.99	44.23 ±19.45	42.87 ±13.86
High School And Above	58.34 ±17.23	48.26 ±15.77	53.85 ±25.45	44.23 ±16.29
p-value	0.01^*^	0.96	0.03^*^	0.85
Occupational Status				
Self Employed	55.90 ±14.06	51.04 ±14.09	49.24 ±19.35	45.09 ±13.04
Never Worked	47.36 ±12.17	41.24 ±12.66	37.53 ±21.58	37.91 ±14.97
Unemployed	52.29 ±11.86	48.74 ±12.45	48.83 ±15.66	43.49 ±12.68
Pensioner	46.94 ±13.19	43.62 ±12.86	41.96 ±19.24	39.32 ±11.82
p-value	<0.001^*^	<0.001^*^	<0.001^*^	0.020^*^
Socio-economic status scale (According to BG Prasad 2014)				
Upper Class	42.44 ±10.11	39.11 ±14.97	34.44 ±18.41	33.56 ±15.64
Upper Middle Class	50.37 ±10.54	47.75 ±10.23	44.67 ±19.78	43.76 ±10.35
Middle Class	55.65 ±13.45	50.37 ±14.53	45.72 ±14.43	43.88 ±16.45
Lower Middle Class	56.29 ±12.86	50.33 ±13.77	50.24 ±19.02	43.67 ±12.83
Lower Class	52.51 ±14.34	47.68 ±14.51	46.10 ±18.17	44.23 ±13.27
p-value	0.004^*^	0.02^*^	0.03^*^	0.03^*^

## Discussion

In the current study, among the sampled 270 elderly residing in urban slums, the total QoL score was 47.52 ± 15.06, showing that the average population had a fair QoL. The physical domain score was 52.74±13.36, the highest among the four domains, while the environmental domain score was the lowest (43.15 ±13.63). In a study done by Venu et al., the social domain score was the highest (69.4 ± 9.7), with the environmental domain being the lowest [11]. This difference in higher scores in the current study in the physical domain can be explained by the larger proportion of people in the 60 to 69 age group, while in Venu et al.'s study, the age group of 65 to 70 comprised the majority. The QoL scores decrease with increasing age, indicating that aging greatly affects the QoL. This situation also prevails in other countries, where similar results are seen in the study conducted by Figueira in Brazil, where the young-old (60 to 69 years) had better QoL scores than the old-old (70 to 79 years) and the oldest-old (80 and above) [12].

Females outnumbered males in the present study; similar findings were noted in other studies [11,13,14]. Females had a lower QoL score in the physical as well as social domains as compared to males; similar findings were reported by Venu et al. [11]. The female gender, although outnumbering the male in proportion in all studies, has poorer QoL scores. These findings need to be further researched so that interventions can be undertaken to promote health and better QoL in females.

Hindus had a better physical domain score but lower social and environmental domain scores in comparison to their Muslim counterparts. Regarding the association of caste with QoL scores, although those of general caste reported higher physical and psychological domain scores, it was not found to be statistically significant. The elderly residing in joint families had a higher QoL score in all four domains. It may be that with increasing age, a support system of family helps them have a better QoL. Similar results were seen among the rural elderly in Haryana [6].

Persons who were either single or widow/widower/separated were seen to have lower QoL scores in physical, social, and environmental domains. The relation between marital status and the well-being of the elderly has been widely studied, especially in western societies. These studies have shown that widowed elderly people have poorer health and well-being than those who are currently married. The divorced appear to be the least healthy, followed by widowed and single elders, and the married appear to be the most healthy. Thus, marital status is one of the key variables in determining their QoL [15, 16]. Various researchers have examined the effects of living arrangements on the QoL of the elderly. According to them, the changes in living arrangements and family structure have a great impact on their physical and psychological well-being [17].

Increasing levels of literacy had a better QoL score among the elderly. Similar patterns were seen in other studies conducted for both rural and urban elderly populations in India [6, 11]. Education has a positive impact on life in general, but further in-depth studies need to be undertaken to draw a causal association from this inference.

This study, being a cross-sectional one, lacks the ability to establish a causal association between the various factors and QoL. This is inherent in the nature of all cross-sectional studies. The present study identified factors such as increasing age, female gender, living alone, living in nuclear families, and illiteracy to be associated with poor QoL scores. With these findings, the authors recommend longitudinal studies to investigate these cause-effect relationships. Since QoL scores are based on self-reported statements, under-reporting or over-reporting, i.e., reporting bias, cannot be ruled out.

## Conclusions

The QoL, although expressed by a number of domains, is inter-related; hence, the concept of QOL should be considered as a dynamic web of intertwined domains. The current study's findings show a decreasing QoL with increasing age. Aging is associated with a decrease in social and economic participation. In areas of compromised living conditions, like slums, the environmental domain contributes to the decrease in quality along with the other domains. Employment status, socio-economic class, and female sex had significantly lower QoL scores in all four domains. Aging and detoriation of QoL and associated problems are not properly understood in the Indian slum population due to the non-availability of relevant data. This study was an attempt to probe and analyze the various problems of the sampled elderly population, with an emphasis on their QoL in various dimensions. The identified factors associated with various dimensions of QoL scores need to be addressed, thereby improving their living habitats.
